# Online interviewing with interpreters in humanitarian contexts

**DOI:** 10.1080/17482631.2018.1444887

**Published:** 2018-03-13

**Authors:** Anna Chiumento, Laura Machin, Atif Rahman, Lucy Frith

**Affiliations:** a Institute of Psychology, Health and Society, University of Liverpool, Liverpool, UK; b Lancaster University, Lancaster, UK

**Keywords:** Qualitative interviews, online interviews, Internet communication, research methods, cross-cultural, humanitarian emergencies, post-conflict, research ethics

## Abstract

**Purpose:** Recognising that one way to address the logistical and safety considerations of research conducted in humanitarian emergencies is to use internet communication technologies to facilitate interviews online, this article explores some practical and methodological considerations inherent to qualitative online interviewing. **Method:** Reflections from a case study of a multi-site research project conducted in post-conflict countries are presented.  Synchronous online cross-language qualitative interviews were conducted in one country.  Although only a small proportion of interviews were conducted online (six out of 35), it remains important to critically consider the impact upon data produced in this way. **Results:** A range of practical and methodological considerations are discussed, illustrated with examples.  Results suggest that whilst online interviewing has methodological and ethical potential and versatility, there are inherent practical challenges in settings with poor internet and electricity infrastructure.  Notable methodological limitations include barriers to building rapport due to partial visual and non-visual cues, and difficulties interpreting pauses or silences. **Conclusions:** Drawing upon experiences in this case study, strategies for managing the practical and methodological limitations of online interviewing are suggested, alongside recommendations for supporting future research practice.  These are intended to act as a springboard for further reflection, and operate alongside other conceptual frameworks for online interviewing.

## Introduction

Unstable settings, such as those where humanitarian emergencies occur, give rise to logistical considerations when designing and planning research, including restricted access to sites and populations (Karray, Coq, & Bouteyre, 2017). One way to overcome access difficulties is to use Internet communication technologies; for example, online interviewing. In choosing to integrate this solution into research, practical and methodological implications must be considered. When the interview process involves an interpreter, these considerations, and the accompanying methodological implications, increase in complexity.

**Table 1.** Key characteristic of interviews conducted online.

Interviewee*	Participant gender	Participant and researcher prior relationship	Interpreter involved	Interview location	Description of connection quality
Leslie (C3, I1)	Female	Yes	Yes	Hospital	Call repeatedly dropped. Switching between Adobe Connect™ and Skype™ throughout interview.
Mollee (C3, I2)	Female	No	Yes	Hospital	Some sections of overlapping speech, conducted in Adobe Connect™.
Fernanda (C3, I3)	Female	No	Yes	Hospital	Interviews conducted in Adobe Connect™, experienced multiple fade-outs of speech on both sides of conversation.
Shaheen (C3, I6)	Male	Yes	No	Hospital	Interviews conducted using Adobe Connect™ over two sessions due to power cut. No problems during interviews.
Margareta (C3, I7)	Female	No	Yes	Home	Unable to use Adobe Connect™, used Skype™ with video. Power cut led to switching from computer to skype for mobile to continue the conversation.
Tanika (C3, I8)	Female	Yes	Yes	Hospital	Interview conducted using Skype™ as Adobe Connect™ unable to connect. Repeated dropping of calls at beginning of interview.

*All names are pseudonyms allocated by the researcher, ensuring the protection of participant anonymity.

**Table 2.** Logistical and methodological recommendations for managing online research interviews.

Logistical/methodological consideration		Suggested strategies to manage/account for these, drawing upon experiences in this study
*Social construction of interview space*	Internet, electricity and, where applicable, Internet-enabled mobile phone infrastructure.	– Discuss strengths and weaknesses of local infrastructure with participants/contacts based in the participants’ setting. – Use this information to plan an interview schedule that is feasible and flexible within the identified constraints—for example, would three shorter interviews be preferable to one longer interview if infrastructure is highly unreliable?
	Researcher and participants’ prior familiarity with online synchronous interviews, including software to be used for interviews.	– Gather information from participants about their previous use of online interviewing platforms, including the one to be used for interviews. – Researcher reflects upon their own familiarity and comfort with online interviewing technology, and the impact this may have upon their interviewing style. – Develop instructions to share with participants in advance of the interview on how to establish a connection and use the interview software. – Account for the time required to set up connections before interviews commence.
*Rapport building*	Prior relationships between researcher and participant	– Consider how prior relationships will set up expectations of the interview encounter; in particular, power relations and role performance. – What tools are available for the participant to “meet” the researcher and vice versa (i.e., use of video; photo icons, etc.) – Provide participants with a verbal overview of the intended progress of interviews, and any requests such as that the participant speak slowly and clearly.
	Presence of third parties (interpreters/chaperones)	– Ensure expectations about the role of any third party are established and agreed at the interview outset.
*Role performance*	Accounting for the interview environment	– As in any fieldwork, field notes are an essential tool to support contextual interpretation and analysis of interviews. – For online interviews, including observations about the setting in which the researcher and participant are based is essential, including interruptions or presence of others in the participant’s setting. – Documenting facial expressions or hand gestures can aid understanding emphasis of a point or confusion over a question.
*Disembodiment*	Managing silences	– Inform the participant during the interview overview of how non-verbal actions will be communicated (i.e., when writing field notes). – Consider all interview parties providing “stage directions” to narrate what is occurring during pauses in conversation.
	Inaudible segments	– As the researcher, be prepared to ask the same question in different ways to avoid excessive repetition should connection problems cause difficulty in the participant hearing a question. – Use techniques such as reflecting back or incorporating language used by the participant to demonstrate responses have been heard, as well as to ensure correct understanding.
*Reflection*	Asking for participants’ experience of the online interview format	Asking participants for reflections on their experience of the online interview format can provide important feedback to (a) improve future interactions; and (b) complement field notes about a participant’s level of engagement during the interview.

The reflections, in the form of a case study, presented in this article are drawn from the lead author’s (AC) experience of conducting online cross-language qualitative interviews as part of a multi-site study. Incorporating online synchronous interviews was necessary due to security considerations preventing travel to one of the research sites to conduct in-person interviews. Although in this multi-site case study only a small proportion of interviews were conducted online, the impact upon data produced in this way needs to be critically considered. To this end, a range of practical and methodological considerations are identified and discussed, illustrated with examples and quotes. This small case study of online interviews is valuable for the methodological reflections and learning that can be applied and extended through further research. The reflections and considerations raised in this article are intended to complement wider literature that presents in-depth discussions of the methodological, practical and theoretical issues presented when conducting social science research online (Hine, 2012; Salmons, 2015; Snee, Hine, Morey, Roberts, & Watson, 2016). Specifically, presenting reflections from research conducted in a post-conflict setting complements this literature by considering some key methodological issues in a situated research context.

## Case study: researchers’ construction and management of ethical issues in post-conflict mental health research

The case study from which this article is drawn empirically examines how “ethics” is defined, understood, applied and managed by mental health researchers working in post-conflict settings, focusing upon the interaction between constructions of procedural and in-practice ethics (Guillemin & Gillam, 2004). It is a qualitative multi-site study (Yin, 2009), involving interviews with mental health researchers in three post-conflict settings in South Asia. A phenomenological orientation was adopted to emphasize the lived-through quality of researchers’ experiences of ethics (Schutz, 1945). The research aims to produce findings relevant to the conduct of ethical mental health research in post-conflict, and by extension humanitarian, contexts.

Between September 2014 and February 2015, 35 individual in-depth interviews were conducted across 3 South Asian countries and in the UK. All interviews were led by the researcher (AC), with an interpreter involved when required. Interviews followed a semi-structured topic guide that explored participants’ perspectives of the construction and management of procedural and in-practice ethics (Guillemin & Gillam, 2004), complemented by remaining open to iterative evolution of interview topics to explore both within and between countries (Rapley, 2007; Yin, 2009).

Twenty-five interviews were conducted with interpreters, with the remaining participants choosing to speak in English. All interpreters were hired in-country following selection interviews, with attention paid to participants’ preferred languages. This article will focus upon six interviews that were conducted online in the third country, of which five involved an interpreter (see Table 1). The focus of this case study is the interview format and methodological and practical considerations this posed for qualitative interviews; therefore, the small number of interviews is deemed sufficient for these considerations to be explored. Attention is focused on the online interview format, with the additional complexities of interviewing through interpreters discussed where relevant. For an in-depth discussion of the methodological approach this study took to integrating interpreters, see Chiumento, Rahman, Machin, and Frith (2017).

### Setting description

Research was conducted in three countries in South Asia, with a 1-month period of data collection in each. All three countries have recent histories of conflict and disaster which meant the in-country Internet and electricity infrastructures were poor, particularly in rural sites, including bandwidth limitations and unpredictable power cuts. In the third country, the security context deteriorated in the weeks leading up to planned fieldwork, which necessitated the adaptation of interview formats to include online synchronous interviewing via Adobe Connect™ or Skype™.

### Ethical oversight

Ethical approval was obtained from each South Asian country and the University of Liverpool. Online interviewing had been outlined as a possibility in the original ethical applications, recognizing the potential instability of the research settings. When confirmed for Country 3, additional information on the online interview format and processes (i.e., addressing confidentiality) was approved by the relevant country and Liverpool ethical review committees. All participants provided voluntary written informed consent prior to interviews. To protect confidentiality, data has been fully anonymized. This includes the use of pseudonyms for each participant, and the replacement of all potentially identifying information with fictitious country/place/organization names, denoted by {} brackets.

## Online interviewing

The physical separation of researcher and interpreter from the participant raises both practical and methodological considerations. To ensure transparency regarding the reliability and rigour of qualitative interviews, it is important to explore the consistency of online and offline interviewing with underlying research epistemology, application of methods, and how these ensure the desired research outcomes are attained (James & Busher, 2009).

To manage the shift in interviewing format, a brief review of literature was conducted prior to data collection to achieve methodological acculturation (Kovats-Bernat, 2002). Benefits of online interviewing were highlighted, including: limited ecological impact as compared to in-person interviews (Hanna, 2012); reduced time commitment due to eliminating travel (Deakin & Wakefield, 2014); and the increasing spread and advancement of technologies that make online interviews convenient and cost-effective (Deakin & Wakefield, 2014; Sullivan, 2012). Limitations were that these benefits require prerequisites of high-speed Internet access and computer literacy of all parties (Janghorban, Roudsari, & Taghipour, 2014). Additionally, potential technical challenges include sound quality or webcam issues, a time-lag in the audio/video feed meaning sound and/or video is relayed slower than real time, and potentially lost data as a result of technological failure (Saumure & Given, n.d.; Sullivan, 2012). Drawing upon reflections of the researcher, interpreter and participants documented during fieldwork, this article will critically engage with the methodological and practical considerations that need to be addressed when conducting online interviews.

### Social construction of online interview spaces

In social science research, emphasis is placed upon the importance of the field, both epistemologically and methodologically, as a space where researchers and participants engage in the act of research (Clifford, 1997). When conducting research online, the *site* of research is displaced and the *sight* between researcher and participant interrupted (James & Busher, 2009).

In this study, the researcher and interpreter were based in the capital city, whilst participants were in a city in a different region. Being based at a non-governmental research institution in the capital city meant the researcher had access to strong Internet infrastructure, including multiple Internet networks and a back-up generator for when the electricity supply ceased. By comparison, five participants conducted interviews from a governmental hospital with poor Internet infrastructure, physically located in a shared office—with associated interruptions and privacy limitations. The sixth participant conducted the interview from her home in a private room, with the only interruption being a family member bringing snacks. Power cuts affected the participants’ home and hospital settings, with implications for 2 interviews: a computer running out of battery that required rescheduling an interview to continue 14 days later; and a participant switching to a mobile device to continue the interview after computer batteries had run out.

By conducting interviews via Adobe Connect™, the researcher was able to consciously construct a research environment for the study. Adobe Connect™ is subscription-based specialist web conferencing software frequently used in academic contexts. It has features to allow recording of video and audio within the software, with access to the meeting space and recordings password protected to ensure confidential information is safeguarded. It is for this reason that this platform was preferred to other options including Skype™ where the privacy of information cannot be guaranteed. Therefore, in choosing to use Adobe Connect™ for interviews the researcher was in a position of power, consciously constructing a professional site of research that afforded privacy protections and recording capabilities to facilitate the act of research (James & Busher, 2009).

It is important to note that AC is familiar with the use of online communication tools including regular use of Skype™ for meetings as well as personal use, and has used the Adobe Connect™ platform for teaching and meetings, ensuring familiarity with its features for application to this study. All research participants mentioned their familiarity with online platforms—notably Skype™—for both professional and personal communication. However, to the researcher’s knowledge only one participant had prior experience of Adobe Connect™. This lack of familiarity meant that for interviews conducted at the hospital a participant supporting the study’s logistical arrangements and oriented to Adobe Connect™ by the researcher prior to study commencement was present at the start of each interview to set up the online space. Similarly, for the participant at home, the researcher provided guidance on setting up the connection prior to commencing the interview. This process of establishing a connection and introducing participants to the online space lasted around 10 minutes. Despite these brief orientations, there were instances during interviews where unfamiliarity with the software led to accidental muting of the microphone:

**R: We have lost your sound hang on one second. Ah, you’ve been muted, hang on (.5) (Ask her)**

I: #37.24–37.28#

P: #37.29–37.30#

**R: (.3) Oh you’ve muted it again, hang on, I’ll unmute it.**

I: #37.38–37.41#

**R: (.2) ((to I)) I can do it for her, ((to P)) I don’t want to do it and then you. (.8) Okay I can hear you, oh no hang on, don’t touch anything, let me do it.**

I: #38.00–38.02#

**R: (.7) You’re touching it, so you’re doing it at the same time. Okay we seem to have fixed it. Okay ((R laughs)), sorry.**

*Fernanda (C3, I3)*^1^

Consequently, whilst the researcher, interpreter and participants all had prior experience of online synchronous communication technologies for professional and/or personal purposes, the use of Adobe Connect™ put the researcher in a position of power due to her familiarity with the software. Equally, to the researcher’s knowledge none of the participants had engaged in interviewing or being interviewed via online platforms, and neither the researcher nor the interpreter had conducted qualitative interviews online before. Therefore, in engaging in online interviews the researcher, participant, and interpreter were drawing upon personal and professional micro-cultures that shape understandings of the personal and professional use of online communication tools (James & Busher, 2009). In addition to these prior experiences, to prepare for interviews a number of steps were taken, including interpreter training which involved the conduct of in-person interview role plays with dummy participants (see Chiumento et al., 2017, for a discussion of interpreter training). Furthermore, an introduction to the Adobe Connect™ platform was provided first through briefly testing the software with a colleague based in another room in the office hosting the researcher, and secondly through a group informed consent session conducted prior to interviews with all participants to be interviewed online. This informed consent session provided all parties with an introduction to the online format, as well as providing participants with an introduction to the mediation of conversations by an interpreter.

The flexibility of online interviewing did facilitate the inclusion of one participant in the study because she was able to participate from home, meaning she could arrange the interview around other commitments. In this case, and in the renegotiation of the timing of interviews conducted from the hospitals, elements of the site of power between researcher and participant shifted to the participants who negotiated the timing and location of interviews around existing commitments. When compared to in-person interviews in other countries, it was felt that the online format made the adjustment of prearranged interview timing more likely than with in-person interviews. Similar experiences have been documented by other researchers who note that participants may feel less obliged to adhere to pre-agreed timings online than in person (Holt, 2010). The fluidity of the physical interview site and associated power dynamics will continue to evolve as technology and smartphones advance (Botha et al., 2010), and is particularly relevant to conducting interviews in inaccessible locations such as after a humanitarian emergency.

### Maintaining confidentiality/privacy

Two facets of the concepts of confidentiality and privacy will be discussed; the first relates to the researcher’s ethical obligation to ensure the confidentiality of information shared via an online site of research conduct; and the second relates to privacy of conversations when the researcher has no control over the location from which participants conduct interviews (British Psychological Society, 2017).

Conscious construction of an online secure password-protected site ensured the researcher was able to achieve her ethical responsibility to ensure the privacy of information exchanged online (British Psychological Society, 2017). Critically, the choice to use Adobe Connect™ sought to minimize the risk of harm to participants by ensuring researcher and participant control over access to confidential data. Ess and The Association of Internet Researchers (AoIR) (2004) argue that consciously establishing a “safe” online environment can act to encourage participant disclosure in interviews. Equally, prior relationships between researchers and participants play a role in shaping trust, underpinned by a sense of the researchers integrity towards the protection of confidentiality and anonymity (Ess & The Association of Internet Researchers [AoIR], 2004; James & Busher, 2009). In this study, trust in the researcher’s conduct was felt to have been established through prior relationships with some participants, which had led to internal narratives about who AC was, alongside perceptions of how a researcher conducting a study into research ethics would behave. This projection of the researcher as prioritizing participant privacy is reinforced in the following text conversation when discussing a participant’s request to switch from Adobe Connect™ to Skype™ to continue an interview:

**R: The one thing to note with skype is I cannot guarantee the confidentiality of the conversation—skype have the right to record it if they want to.**

Tanika (C3, I8)

This change in software occurred in a number of interviews, most frequently at the request of participants. Therefore, in proposing the use of Skype™, the agency of the participant to make an informed choice about the levels of privacy and security they are comfortable with is apparent (Ayling & Mewse, 2009).

Another issue encountered was the privacy of the site from which interviews were conducted. The researcher was able to ensure a private room from which she and the interpreter conducted interviews. Conversely, due to the online format, the researcher is unable to control the participant’s environment to ensure confidentiality. In this study, due to a lack of alternative options, for the majority of interviews participants were located in a shared office in a hospital. As experienced by other researchers, the lack of control over the physical setting in which participants were located led to interruptions or the presence of others in the background (Deakin & Wakefield, 2014). The impossibility of knowing when people were/were not present during interviews could lead to the misinterpretation of visual cues, such as smiles or turning of heads, which could be non-verbal cues relating to the conversation, or a response to the presence of others in the room (Seitz, 2016).

In an attempt to enhance the privacy of conversations, participants were encouraged to use earphones so only their responses could be overheard by others who may also be present in the shared office. Despite this, it is possible participants self-censored their responses for fear of saying the “wrong” thing in front of colleagues, which is likely to have impacted upon the depth of interview data.

### Role performance

When interviewing, the social roles of those engaged in the interaction—in this case the researcher, interpreter and participant—are negotiated in a social setting in which the various performers engage in impression management (Goffman, 1959). Sullivan (2012) argued that synchronous online environments are able to satisfy Goffman’s (1959) criteria for assessing impression management including visual non-verbal cues such as smiles, frowns, shrugs, etc., and paralinguistics such as stressing words or sighing. In this study, the research participants were researchers who brought their own understanding of the norms governing an interview encounter, including perceptions of the behaviour of a “good participant” (Frisoli, 2010; Wengraf, 2001), such as ensuring full attention to the interview and articulating their responses to questions as clearly as possible. Given that the quality and depth of qualitative interviews depend to a certain extent upon the relationship and rapport between the interviewer and participant built in part through non-verbal cues (Salmons, 2015), it is important to consider the impact of the availability/unavailability of non-verbal cues as a result of the online interviewing format. In the context of this study it was found that non-verbal cues signalled participants’ responses to the direction of questions. These included, for example, eye rolling or hand gestures to signal exasperation or frustration at requests to clarify taken-for-granted aspects of the social and cultural milieu, or smiles and nodding to indicate agreement with a line of questioning or confirming the researcher’s understanding of a point.

In capturing the projection of non-verbal cues a number of limitations were encountered. Low bandwidth meant even when available, visual cues were limited or froze, and a time-lag in relaying the audio meant such cues were asynchoronous to verbal utterances. Additionally, even when available, video restricted physical presence by only displaying the participant’s head and shoulders (Seitz, 2016), leaving absent other body language such as positioning of hands and legs. Furthermore, simple non-verbal connections, such as eye contact, are impossible in online formats (Seitz, 2016).

To ensure transparency regarding this potential limitation, the researcher maintained notes in her research diary regarding perceptions of what was happening in the environment around the participant; for example, “Participant looking at someone else in room and shaking head in response to a question/comment from them” (C3, I2), or “Door opens in room P is in, can see her eyes go up to see who is coming in. Some background talking, then door opens and closes again—assume they left the room” (C3, I2). These were kept alongside general reflections about the interview environment from the researcher and interpreter, documented immediately after each interview.

Due to the impossibility of predicting connection quality in advance of interviews, flexibility in responding to the availability/unavailability of video was necessary. Once interviews move online, the ability to project and negotiate role performance is restricted, particularly in the absence of video. This includes limited access to cues regarding background demographics such as age; self-presentation—for example, though clothing; and subtle cues such as smiling, frowning or nodding. Additionally, in the context of this cross-cultural study conducted in South Asia, the availability of facial expressions could not be assumed as the research participants’ cultures include females wearing veils that cover their face. This impacted upon the availability of non-verbal cues such as smiles, and occasionally the clarity of verbal utterances.

When working in cross-cultural contexts, restricted visual cues, coupled with the involvement of an interpreter, reinforced the distance between the white, Western, English-speaking researcher and local interpreter and participants. This was reflected by participants who commented on the advantages of being able to see the researcher, to “meet” whom they were talking to:

I: … *the video conversation is very important because she wanted to know that who is Anna and how she looks like that er, that who is involved in {Rudo} programme so she just wanted to meet you so that’s like. It’s good.*

Fernanda (C3, I3)

This quote illustrates the importance the participant attached to “meeting” the researcher, emphasizing the desire for in-person interaction. Whilst the extent to which this is achieved via the online format remains limited, it does offers a substitute to in-person interactions where required.

### Rapport building

The researcher had prior relationships with some participants that were felt to aid online interviews, allowing the researcher and participant to build upon previous interaction dynamics. Furthermore, conducting the group informed consent session had enabled all participants to be introduced to the researcher and interpreter in advance of interviews. Having a prior relationship with the researcher, alongside familiarity and comfort with online formats, was identified by a participant as a key factor in influencing the extent to which video supported rapport building and facilitated interview conduct:

P: … actually it depends upon the person … how much another person is comfortable while dealing with a new person …. [N]ormal level of anxiety is definitely there.

Leslie (C3, I1)

As this indicates, a range of factors affect building relationships between researcher and participant. Of the interviews conducted in the third country, six were online and two were in person. When reflecting upon the difference between the online and in-person interviews with participants the researcher had not met before, the suggestion that being comfortable with interacting with someone new is more influential than the interview format is supported. However, it is difficult to isolate factors that may have influenced this. It is possible the gender difference between researcher and participant may have been the critical factor influencing rapport building because the two in-person interviews were with males.

In interviews conducted with an interpreter, the presence of an additional unknown third party may also have impacted upon rapport building, as conversations and therefore connections between the researcher and participant are mediated by a third party. In this country, the interpreter was male whilst the majority of participants interviewed online were female; therefore, patriarchal gender norms in the setting may potentially have influenced narratives. Equally, the researcher found that the relationship with a male interpreter led to a different style of interviewing than was experienced in the other countries when interviewing with female interpreters. It is accepted that the impact of gender norms and interaction dynamics between the researcher and interpreter may in turn have impacted upon efforts to build rapport with participants who may have sensed an awkwardness to the researcher/interpreter relationship. Therefore, the relationship between the researcher and interpreter may also have influenced rapport-building between the participant and researcher/interpreter dyad.^2^ All of these factors may have influenced rapport-building, and further research to explore the role of each is recommended.

### Disembodied interview

Online interviews without video have been characterized as disembodied, with the removal of non-verbal cues acting to limit interview contextualization and potentially reduce the impact of the interviewer on the interview encounter (O’Connor, Madge, & Shaw, 2008). In this study, disembodiment led to a more rushed interview flow, with a diminished emphasis upon rapport talk in favour of report talk (Wengraf, 2001). Interviews were also shorter, despite the online format requiring more time than in-person interviews as a result of the conversation time-lag and additional level of clarification to ensure meaning had been understood. For example, after the first online interview, the researcher reflected that she felt she was unable to draw upon notes taken during the interview to consider the next question, with pressure heightened due to the lack of video. The result was an interview that involved question-and-answer exchanges, rather than an evolving discussion in which probes were organically pursued. This was felt to result from a sense that participants were waiting in anticipation for the next question, and was compounded by the lack of a clear visual connection between the researcher/interpreter and participant in which pauses accompanied by a smile or note-taking can be taken as a cue to embellish or clarify response to a question. Therefore, this disembodiment led to a void between the researcher and participant that the researcher became concerned to “fill”, something others have reported when conducting online text interviews (Markham, 2004).

In an attempt to address this rush to the next question, the expectation of pauses in conversation was established at the outset of the interview when the researcher explained: R: **“I have a notebook, {Interpreter} has the same ((both hold up notebooks to camera)), so we will probably take notes whilst you’re talking, so if you see us looking down that’s what we are doing”** (*Tanika* C3, I8). Furthermore, the researcher in subsequent interviews narrated what was happening during silences or pauses—including when the video was on. For example:

**R: … Um, you’ve given me so many extra questions I want to ask you, er just give me a second to have a think.**

P: Okay.

Margareta (C3, I7)

The researcher would also clarify when the interpreter was finishing writing notes prior to translating what a participant had said:

P: #53:47-55:32#

**R: (.4) Okay he’s just finishing writing.**

P: Okay.

**R: Okay.**

I: She said that ….

Tanika (C3, I8)

This approach was effective in providing the space for more considered questioning and probing. Despite these efforts, the length and frequency of pauses as well as the depth of probing were felt to be less than occurred in in-person interviews, where the researcher can sense how comfortable a participant is with natural pauses in conversation. This approach also increased the sense of interview as performance, with the researcher providing cues akin to stage directions to ensure the participant remained informed about interactions that were out of sight. Whilst these responses were effective in this study, it is noted that, where possible, researchers should conduct practice interviews to explore and select their preferred online interviewing platform, as well as to rehearse strategies to manage foreseeable differences in the experience of online interviewing, such as the unavailability of non-verbal cues and impact of interview disembodiment (Hine, 2012; Salmons, 2015).

### Interview practicalities

When interviewing with an interpreter, the time required for interviews necessarily increases, with interviews across the 3 countries involved in this study lasting on average 90 minutes. Online interviews brought additional considerations that impacted upon interview length, chiefly setting up the conversation at the outset, and interruptions to audio such as fading out or overlapping speech. When recording conversation within the Adobe Connect™ platform, as a result of the time-lag overlapping speech was a significant problem, leading to some lost sections of interview data where it is impossible to distinguish what is being said. By listening back to check recording quality this issue was quickly identified and addressed by using a Dictaphone to double record interviews.

Within the language-processing loop it is recognized that meaning can be lost, misheard or misinterpreted (Frisoli, 2010). Difficulties conveying meaning can be compounded due to technological issues, in this study often resulting in repeated attempts to explain or clarify questions:

P: Um (.2) then the er (.3) consent, confidentiality, er patient comfort. I mean all these are everything.

**R**: **Patient comfort, what does that mean?**

P: Yeah.

**R**: **What does patient comfort mean?**

P: Sorry?

**R**: **What do you mean by patient comfort?**

P: It means that er patient

Tanika (C3, I8)

As a result of these difficulties, for all interviews conducted online the researcher had a heightened awareness of timing than with in-person interviews. For example, one interview conducted online involved 22 minutes of recording in Adobe Connect™, during which 8 minutes of conversation took place; followed by a 1-minute 15 Skype™ conversation before the connection went; and finally a 51-minute conversation in Adobe Connect™. At the end of this interview the participant reflected upon the frustrations that could arise as a result of technological difficulties:

**R: … how you found it in relation to the, the online setup?**

P: Er actually I’m used to it before also but er sometimes, just like today a little exhausting because of the Internet connection.

Leslie (C3, I1)

The potential for frustration due to repeated connection issues led to a concern to keep interviews shorter both to limit the burden upon the participant and to limit interpreter fatigue and potential impact upon translation quality. This resulted in interviews conducted online being shorter and therefore more limited in their depth than those conducted in person.

### Concept of safety

Physical safety is contested in unstable and unpredictable research environments (Hanna, 2012). In this study, whilst both the researcher and participants had opted for online interviews to increase safety and protect all parties from the risks presented by travel, this did not mean participants, in particular, were in a place where they were protected from potential safety threats.

This asymmetry in the relative safety of the researcher and interpreter versus that of the participant brings an additional dimension to the site of interviews (Karray et al., 2017) that carries ethical implications (British Psychological Society, 2017). Notably, it raises a question around the first principle of ethical research practice—the protection of participants from harm (National Commission for the Protection of Human Subjects of Biomedical and Behavioural Research, 1979)—as it can be the very inability to ensure a secure setting for interviews that leads to online interviews in the first place. When working in unstable contexts it has been highlighted that the researcher cannot always be expected to work in safety and security, with each of these concepts framed by knowledge of what constitutes danger in a given site (Kovats-Bernat, 2002). In this study, the decision to conduct interviews online was part of a co-produced approach to protection arrived at by the researcher and an organisational representative from the research site, with local knowledge and advice prioritized when making decisions about fieldwork conduct. As one participant noted:

P: … um keeping in mind the availability and our own problems etcetera. So at times this kind of interaction is also okay.

Leslie (C3, I8)

This construction, normalizing an unstable context as “our own problems”, frequently arose during interviews that considered the impact post-conflict settings may have upon the application of ethics. In settings that are unstable, the concept of researchers protecting participants becomes less applicable, with the assumptions of ideal field sites where researchers are the ones in a position of control no longer holding true (Kovats-Bernat, 2002). Equally, the above quote highlights the appropriateness of online interviewing as an alternative format when the “ideal” of in-person interviewing becomes impossible.

## Methodological considerations for managing online interviews

In this section, suggestions for managing key logistical and methodological considerations that arise when conducting interviews online will be made, drawing upon experiences in this case study (see Table 2). These seek to address the lack of a precedent for online interviewing upon which researchers can build, and avoiding the imposition of in-person interviewing standards upon online interactions (Hine, 2004, 2012). Given the limited number of interviews on which these suggestions are based, they are intended to act as a springboard for further methodological, and practical, reflection, and operate alongside other conceptual frameworks for online interviewing, such as that proposed by Salmons (2015).

In order to validate or refine these suggestions, continued documentation and sharing of experiences of conducting interviews online is encouraged, supporting future researchers who choose this interview format (Ferrante et al., 2015).

## Conclusion

As a result of the shift to online interviewing, this study entailed methodological messiness where the researcher was learning the research process alongside generating data (Rossman & Rallis, 2003). This study views methodology “not as a rigid or fixed framework for the research but, rather, as an elastic, incorporative, integrative and malleable practice” (Kovats-Bernat, 2002, p. 210) that is co-constructed between the researcher, participant and interpreter. In this context, reflexivity towards both the process and outcomes of interviews conducted online is a moral and methodological obligation of the researcher (Frisoli, 2010).

The reflections in this article have identified a range of practical and methodological considerations that arose in the conduct of a cross-language qualitative research study that involved online interviewing. Notably, the challenges of gaining depth of data collected via online interviews are a central consideration when using this interview format.

Online interviewing presents methodological and ethical potential and versatility, but should not be viewed as an easy option (James & Busher, 2009). Through providing practical tips for researchers to implement and evaluate, this article aims to contribute to the development of qualitative research standards specific to online interviews, ensuring the same level of methodological transparency as is expected for in-person interviews. Reflections and feedback on these practical tips are welcomed, as is further research to illuminate considerations such as the role of gender and cultural norms upon building rapport which are touched upon in this study.

## Notes

1. In all interview extracts, R = researcher, P = participant and I = interpreter. When participants are speaking in their native language, this is indicated by a time stamp (e.g., #3.12–3.46#). This was felt to be in line with the studies phenomenological epistemology, ensuring the native spoken word was not written out of transcripts and emphasizing the three-way construction of data involving translation by an interpreter.

2. For a full methodological discussion of the role and impact of the interpreter in interviews conducted in this study, please see Chiumento et al. (2017).

## References

[CIT0001] Ayling R., & Mewse A. J. (2009). Evaluating internet interviews with gay men. *Qualitative Health Research*, 19(4), 566–10. 19299759 10.1177/1049732309332121

[CIT0002] Botha A., Makitla I., Ford M., Fogwill T., Seetharam D., CAbouchabki C., … Oguneye O. (2010). The mobile phone in Africa: Providing services to the masses. Paper presented at the Science real and relevant conference, South Africa Retrieved January 31, 2015, from http://researchspace.csir.co.za/dspace/bitstream/10204/4246/1/Botha_d1_2010.pdf

[CIT0003] British Psychological Society (2017). Ethics guidelines for internet-mediated research. Leicester, UK Retrieved January 15th, 2018 from http://beta.bps.org.uk/sites/beta.bps.org.uk/files/Policy%20-%20Files/Ethics%20Guidelines%20for%20Internet-mediated%20Research%20%282017%29.pdf

[CIT0004] Chiumento A., Rahman A., Machin L., & Frith L. (2017). Mediated research encounters: Methodological considerations in cross-language qualitative interviews. *Qualitative Research, Online Early View*, 1–19. doi:10.1177/1468794117730121

[CIT0005] Clifford J. (1997). *Routes: Travel and translation in the late twentieth century*. Cambridge, MA: Harvard University Press.

[CIT0006] Deakin H., & Wakefield K. (2014). Skype interviewing: Reflections of two PhD researchers. *Qualitative Research*, 14(5), 603–616.

[CIT0007] Ess C., & The Association of Internet Researchers (AoIR) (2004). Ethical decision-making and internet research. Retrieved April 28, 2016, from http://aoir.org/reports/ethics.pdf

[CIT0008] Ferrante J. M., Friedman A., Shaw E. K., Howard J., Cohen D. J., & Shahidi L. (2015). Lessons learned designing and using an online discussion forum for care coordinators in primary care. *Qualitative Health Research*, 26(13), 1851–1861. 10.1177/1049732315609567 PMC4835258 26481942

[CIT0009] Frisoli P. S. J. (2010). Assumptions, Emotions, and Interpretations as ethical moments: Navigating a small-scale cross-cultural online interviewing study. *International Journal of Qualitative Studies in Education (QSE)*, 23(4), 393–405.

[CIT0010] Goffman E. (1959). *The presentation of self in everyday life*. London: Penguin, 1990.

[CIT0011] Guillemin M., & Gillam L. (2004). Ethics, reflexivity, and “Ethically important moments” in research. *Qualitative Inquiry*, 10(2), 261–280.

[CIT0012] Hanna P. (2012). Using internet technologies (such as Skype) as a research medium: A research note. *Qualitative Research*, 12(2), 239–242.

[CIT0013] Hine C. (2004). Social research methods and the Internet: A thematic review. *Sociological Research Online*, 9(2), 10–17.

[CIT0014] Hine C. (2012). *The internet: Understanding qualitative research*. [Electronic book] Oxford: Oxford University Press.

[CIT0015] Holt A. (2010). Using the telephone for narrative interviewing: A research note. *Qualitative Research*, 10(1), 113–121.

[CIT0016] James N., & Busher H. (2009). *Online interviewing*. [Electronic book] Los Angeles, [Calif.]: Sage.

[CIT0017] Janghorban R., Roudsari R. L., & Taghipour A. (2014). Skype interviewing: The new generation of online synchronous interview in qualitative research. *International Journal of Qualitative Studies on Health and Well-Being*, 9(1), 1–3. 10.3402/qhw.v9.24152 PMC3991833 24746247

[CIT0018] Karray A., Coq J.-M., & Bouteyre E. (2017). Delivering online clinical interviews with NGO workers in humanitarian and cross-cultural contexts. *International Perspectives in Psychology: Research, Practice, Consultation*, 6(2), 115–131.

[CIT0019] Kovats-Bernat J. (2002). Negotiating dangerous fields: Pragmatic strategies for fieldwork amid violence and terror. *American Anthropologist*, (1), 208–222.

[CIT0020] Markham A. (2004). The internet as research context In Seale C., Gobo G., Gubrium J. F., & Silverman D. (Eds.), *Qualitative research practice* (pp. 328–344). London: Sage Publications.

[CIT0021] The National Commission for the Protection of Human Subjects of Biomedical and Behavioural Research (1979). Ethical principles and guidelines for the protection of human subjects of research (The Belmont Report). Retrieved December 12, 2017, from http://www.hhs.gov/ohrp/humansubjects/guidance/belmont.html 25951677

[CIT0022] O’Connor H., Madge C., & Shaw R. (2008). Internet-based interviewing In Fielding N., Lee N., & Blank G. (Eds.), *The Sage handbook of online research methods* (pp. 271–289). London: Sage.

[CIT0023] Rapley T. (2007). Interviews In Seale C., Gobo G., Gubrium J. F., & Silverman D. (Eds.), *Qualitative research practice* (pp. 15–34). London: Sage Publications.

[CIT0024] Rossman G. B., & Rallis S. F. (2003). *Learning in the field: An introduction to qualitative research* (2nd ed.). Thousand Oaks, CA: Sage Publications.

[CIT0025] Salmons J. (2015). *Qualitative online interviews: Strategies, design and skills* (2nd ed.). London: Sage.

[CIT0026] Saumure K., & Given L. (n.d.). Using skype as a research tool: Lessons learned from qualitative interviews with distance students in a teacher-librarianship program. Retrieved January 31, 2015, from http://lrsv.umd.edu/abstracts/Saumure_Given.pdf

[CIT0027] Schutz A. (1945). On multiple realities. *Philosophy and Phenomenological Research*, 5, 533–576.

[CIT0028] Seitz S. (2016). Pixilated partnerships, overcoming obstacles in qualitative interviews via Skype: A research note. *Qualitative Research*, 16(2), 229–235.

[CIT0029] Snee H., Hine C., Morey Y., Roberts S., & Watson H. (Eds.). (2016). *Digital methods for social science: An interdisciplinary guide to research innovation*. [Electronic book]. London: Palgrave Macmillan.

[CIT0030] Sullivan J. R. (2012). Skype: An appropriate method of data collection for qualitative interviews? *The Hilltop Review*, 6(1), 54–60.

[CIT0031] Wengraf T. (2001). *Qualitative research interviewing: Biographic narrative and semi-structured methods*. London: Sage.

[CIT0032] Yin R. K. (2009). *Case study research: Design and methods* (4th ed.). Los Angeles, CA: Sage Publications.

